# Tetanus and Its Treatment

**Published:** 1919-03-08

**Authors:** 


### TETANUS AND ITS TREATMENT.

### A War-Time Retrospect.

Following the isolation of the specific bacillus of tetanus by Kitasato in 1889 some years elapsed before several workers, of whom Behring is perhaps the best known, succeeded in producing a high and lasting immunity in animals. This effect was finally obtained by injecting crude toxin attenuated by the addition of a chemical agent such as iodine trichloride, and nowadays, for practical application, horses are almost universally used. Unfortunately, these animals are so sensitive to the toxins that the greatest care is necessary in the first stages of immunisation. After the initial uncertainty, if their blood is found to contain a satisfactory resisting power, the undiluted toxin may be injected in increasing doses, and in this way a serum of very considerable strength obtained. Very early in the war large supplies of antitoxic tetanus serum became available, making both prophylactic and therapeutic measures a working possibility. From time to time have appeared, in association with the names of Sir William Leishman, Sir David Bruce, and others, reports and analyses of the cases occurring with the British forces, and to-day, in the closing stages, inspection of these details gives information of exceptional interest. Tetanus in civil practice could not be described as rare, but insufficient instances appeared before the average worker to give him more than a vague idea of the value of serum treatment, its limitations, and the means of its correct administration. The Army figures demonstrate the situation from many points of view, and it is upon them that these notes are chiefly based.

### The Mode of Infection.

Present in the alimentary canal of animals and gaining access to the ground in the excrement and manure, the baccilli or spores enter the body by any abrasion in skin or mucous membrane, generally remain localised at this point, develop and produce their toxins. These are taken up by peripheral nerve endings and carried along the axis cylinders to the central nervous system. Wound laceration, deficient aeration, and the subsequent exudation appear to favour development, for it is stated that washed tetanus spores may circulate harmlessly in the blood of experimentally inoculated animals. As it occurs in both acute and chronic forms, merging one into the other and showing corresponding variation in death-rate and incubation period, this infection has always been particularly difficult to observe by the statistical method. Experiments suggest that after entrance there comes a time when the toxin is so closely attached to the nerve tissues that no amount of anti-toxin will suffice to withdraw or counteract its effects. Unfortunately this fixation occurs early, even before the manifestation of typical symptoms by which the disease may be recognised. It is impossible to be sure whether the amount of poison taken up is sufficient to cause a fatal termination, and therefore doubtful, if at any particular period, an adequate dose of antitoxin can be relied upon to turn the balance in the patient's favour. Delay is necessarily fatal, and on theoretical grounds, a case should be given the largest dose compatible with safety the moment it comes under observation. This was the view at the outbreak of hostilities, and at that time no brilliant array of cures was expected, but a confident hope fixed upon the possibilities of prophylaxis.

### General War Considerations.

It is not surprising that the first months of fighting saw a high incidence of both tetanus and gas gangrene cases, and the awful conditions prevailing, the lack of sufficient remedies, and the dislocation of all organised medical field work is too well known to require description. The figures of these recent case-analyses bring out some interesting points when the periods are taken consecutively. Taking first the ratio of tetanus cases to the total number of wounded, one sees an abrupt and tremendous fall in November 1914, coinciding with increased supplies of serum and introduction in mid-October of the general custom of prophylactic antitoxin injections. Some interesting charts suggest relations between the weather and the liability to infection. The number of cases is plotted against both rainfall and temperature. Rain itself does not appear to exert much direct influence, for the incidence is lowest during some of the wettest months of the year, but on the other hand temperature may have a more marked effect. Rapid drying of the ground occurs from May onwards, and at this time the number of infections is decidedly low. Periods of fighting and movement bring other factors to bear, but on the whole the seasonal tendency prevails, and generally the case incidence of tetanus has shown a satisfactory fall in the summer and autumn months. Next to consider the types of injury, one finds it very hard to arrive at any definite conclusion. Infection in either body or limbs appears to be equally frequent, and the mortality rate approximately the same. Entrance of the organisms in cases of "trench foot" have been common enough to necessitate an order from the office of the Director-General of Medical Services that prophylactic inoculation was to be given in all cases, and its application became general from early 1917 onwards. As might be expected, the commonest tetanus wounds are extensive, deep, or lacerated, while sepsis and gas gangrene frequently occur as coincident conditions, thus impairing the value of many statistics. One would expect that a wound accessible to cleansing and local treatment and of moderate depth is slightly in favour of the infected man's recovery, but after or before manifestation of symptoms extensive operative treatment or amputa-

**Tetanus and its Treatment**—(*continued*)

tion do not appear to reduce the mortality. In the earlier the figure is given as over 70 per cent. in the case of upper limbs and 60 per cent. for lower, and the record is approximately the same for the later operation. Also, in a table indicating cases in which local operative measures with incision, etc., were practised, there is no definite suggestion of an ultimate saving of life by the procedure.

### Incubation Period.

In every series of cases the death-rate is seen to be highest in those of short incubation period, *e.g.*, ten days and under corresponds to 60 per cent., as compared with 30 per cent. after periods of twenty-five days or more. The average incubation time for non-fatal cases is taken as about sixteen days, with three or four days less for those in which death supervenes, while curves drawn to show proportion of cases at any given incubation period, a large number of infections are seen to become manifest on the seventh or eighth day. The mortality is then even higher than those mentioned above—namely, 70 per cent. or over.

### Prophylaxis and Mortality.

At the time of its introduction and up to July 1917, the most approved prophylactic dose seems to have been about 500 units, and the relatively low incidence during the Flanders fighting of 1915 and the Somme battles in later days gives ample proof of the good effect produced. After this period, however, an order was issued recommending increased doses up to 1,500 units in every typically contaminated wound with or without fracture of bone. The succeeding months are then seen to show not only the lowest case rate of all, but also a definite fall in mortality of the disease. Variable amounts being given by different officers we are told that no reliable comparison of groups receiving intermediate dosage can be given, and it is therefore difficult to say whether 1,500 is the optimum. Then, still later, there is evidence of value in repeated inoculations, and this so definite that the Tetanus Committee of the War Office recommended that every soldier should be given four inoculations at intervals of seven days. Following the adoption of this method the incidence remains extremely low, but one must remember that very many factors may have played a part in those times of more complete medical organisation. Nevertheless, it is noteworthy that in these cases the mortality falls to less than 20 per cent., or less than one-third of the corresponding figure given in the first two years of war. The result would undoubtedly suggest, if we take the view that toxin and anti-toxin reaction is a quantitative process, that a yet greater total dosage may be followed by even more outstanding success.

### Therapeutic Use of Antitoxin.

With regard to the administration of antitoxin after symptoms have appeared, the greater the number of cases compared, the smaller appears to be the difference in value of the possible methods of injection, namely, introthecal, intravenous, subcutaneous and intramuscular. The authors of the most recent analyses remark that the second is open to possible anaphylactic complications, and presents dangers not to be encountered in the other routes, although to obtain rapid effects the risk may be worth incurring. Of the others they do not make definite choice, but suggest that all should be used with any given case in order that the largest possible dose of antitoxin may be made available at the earliest possible moment. Figures fail to show that any one scheme of treatment is more successful than another, but a strong argument in favour of serum therapy is given by tables showing a gradual fall in case mortality as the total amount of toxin is increased. At one end we see that 5,000 to 10,000 units, corresponding to 76 per cent. of fatal cases, at the other 100,000 units, giving an average of only 20 per cent. of deaths. As in prophylaxis, the indication is again strongly towards the use of greater doses, and apparently the chief questions to be answered are those which concern the injection of the maximum quantities of antitoxin compatible with safety.

### Conclusion.

Taking a three-year series of the cases which actually developed the tetanic symptoms, we see a steady decrease in the mortality rate, a general average of 68 per cent. in 1915, compared with 20 per cent. in 1918. Whatever general influences have been brought to bear, most of this improvement is undoubtedly due to serum therapy, and although perhaps not so successful as the corresponding agency in diphtheria, yet the value of tetanus antitoxin in the war can never be overestimated. It may have been at times a disappointment, but the facts roughly quoted above prove it to have been the reverse of a failure, and likely to achieve yet greater success as knowledge improves. The number of men who, without inoculation, would have been infected is undoubtedly tremendous. The obvious lightening of symptoms in those prophylactically injected, the remarkable fall of case incidence in November 1914, and the present-day lowered mortality rate among all reported instances, give some suggestion of an army's debt to the laboratories of science, and the greatest hope for future control of this fatal disease.

